# Efficient recombinant production of mouse-derived cryptdin family peptides by a novel facilitation strategy for inclusion body formation

**DOI:** 10.1186/s12934-023-02016-2

**Published:** 2023-01-13

**Authors:** Yuchi Song, Yi Wang, Shaonan Yan, Kiminori Nakamura, Takashi Kikukawa, Tokiyoshi Ayabe, Tomoyasu Aizawa

**Affiliations:** 1 Laboratory of Protein Science, Graduate School of Life Science, Hokkaido University, Sapporo, Hokkaido Japan; 2 Innate Immunity Laboratory, Graduate School of Life Science, Hokkaido University, Sapporo, Hokkaido Japan; 3 Laboratory of Biological Information Analysis Science, Graduate School of Life Science, Hokkaido University, Sapporo, Hokkaido Japan

**Keywords:** Antimicrobial peptides, *E. coli*, Inclusion bodies, Recombinant production, Cryptdin, Disulfide bonds, Deformylation, Antimicrobial activity

## Abstract

**Background:**

A number of antimicrobial peptides (AMPs) hold promise as new drugs owing to their potent bactericidal activity and because they are often refractory to the development of drug resistance. Cryptdins (Crps) are a family of antimicrobial peptides found in the small intestine of mice, comprising six isoforms containing three sets of disulfide bonds. Although Crp4 is actively being investigated, there have been few studies to date on the other Crp isoforms. A prerequisite for detailed characterization of the other Crp isoforms is establishment of efficient sample preparation methods.

**Results:**

To avoid degradation during recombinant expression of Crps in *E. coli*, co-expression of Crps with the aggregation-prone protein human α-lactalbumin (HLA) was used to promote the formation of stable inclusion bodies. Using this method, the production of Crp4 and Crp6 by the BL21 strain was effective, but the expression of other Crp isoforms was not as efficient. The results of a cell-free system study suggested that Crps were degraded, even though a substantial amounts of Crps were synthesized. Therefore, using the Origami™ B strain, we were able to significantly increase the expression efficiency of Crps by promoting the formation of erroneous intermolecular disulfide bonds between HLA and Crps, thereby promoting protein aggregation and inclusion body formation, which prevented degradation. The various Crp isoforms were successfully refolded in vitro and purified using reversed-phase HPLC. In addition, the yield was further improved by deformylation of formyl-Crps. We measured the antibacterial activity of Crps against both Gram-positive and Gram-negative bacteria. Each Crp isoform exhibited a completely different trend in antimicrobial activity, although conformational analysis by circular dichroism did not reveal any significant steric differences.

**Conclusion:**

In this study, we established a novel and efficient method for the production of the cryptdin family of cysteine-containing antimicrobial peptides. Additionally, we found that there were notable differences in the antibacterial activities of the various Crp family members. The expression system established in this study is expected to provide new insights regarding the mechanisms underlying the different antibacterial activities of the Crp family of peptides.

**Supplementary Information:**

The online version contains supplementary material available at 10.1186/s12934-023-02016-2.

## Introduction

Antimicrobial peptides (AMPs) are the primary factors of innate immunity and are the frontline of biological defense mechanisms against infection [1]. Antimicrobial peptides exhibit antimicrobial action, from a low concentration against Gram-negative bacteria to Gram-positive bacteria, fungi, yeast, spirochetes, protozoa, and ultimately viruses [2, 3]. Most AMPs are positively charged [4, 5] and are thought to exhibit microbicidal activity through a membrane-disrupting mechanism due to their interaction with negatively charged microbial membranes. However, the detailed mechanisms of membrane destruction and other such phenomena remain largely unknown. Recently, the emergence of antibiotic-resistant bacteria has become a limitation in the use of antibiotics [6]. AMPs have attracted considerable attention owing to their potent activity and unique antibacterial mechanisms [7–9].

**Fig. 1:**
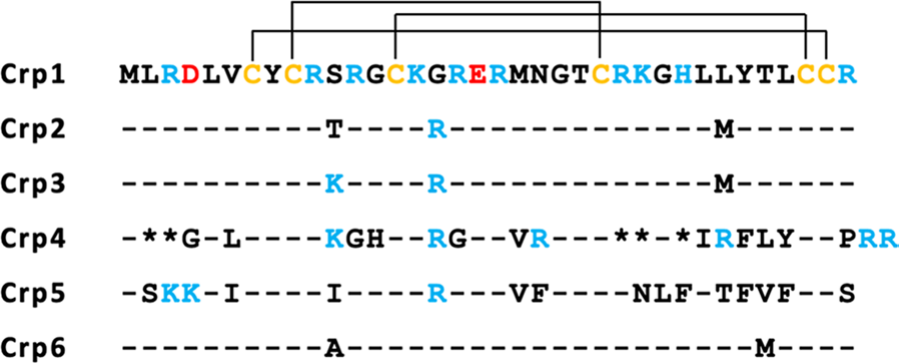
Amino acid sequences of the six Crps. Some amino acid residues are color-coded as follows: Cys, yellow; Arg/Lys, blue; Glu/Asp, red. The three disulfide cross-links (cysteines 1–6, 2–4, 3–5) are also depicted. Dashes in lines of sequence indicate residues of identity with Crp1; and amino acids listed in lines of sequence indicate differences between each Crp and Crp1; asterisks in the Crp4 sequence indicate filler characters introduced to maximize the alignment

Cryptdins (Crps) are a type of AMP, also known as α-defensins, found in mouse intestinal Paneth cells [10]. It has been reported that Crps contribute to the antibacterial barrier function of the small intestinal mucosa, and the selective activity of Crps may be related to the composition of the intestinal microbiota in vivo and homeostasis of the entire intestine [11–13]. Furthermore, low Crp levels are thought to be closely related to dysbiosis, which is an abnormality of the intestinal microflora, and the various diseases it induces. Studies in mice have shown that Crohn’s disease, which is a type of inflammatory bowel disease [14], as well as graft-versus-host disease [15, 16], which is a harmful immune response after bone marrow transplantation, and depression caused by psychological stress [17] are strongly associated with abnormalities in Crp concentration or quality. Similar to other α-defensins, Crps have a characteristic three-stranded β-sheet structure containing six cysteine residues that form three disulfide bonds between Cys1–Cys6, Cys2–Cys4, and Cys3–Cys5 [18]. There are six different isoforms of Crp (Fig. 1; Table 1) [19], and most studies to date have been conducted on Crp4 [20–22] and only a few on other Crps. The level of gene expression of different Crps in various positions in the small intestine differs, and their dissimilar characteristics indicate that different isoforms appear to have specific roles in the small intestine [19, 23–25]. A better understanding of Crps requires them to be produced in a more efficient manner.

**Table 1:** Characteristics of cryptdins

Crps	Mw	PI	GRAVY score	Charge
Crp1	4253.12	9.61	− 0.483	+ 7
Crp2	4384.32	9.86	− 0.647	+ 8
Crp3	4411.39	9.99	− 0.736	+ 9
Crp4	3886.55	9.86	− 0.397	+ 8
Crp5	4339.32	9.86	0.169	+ 8
Crp6	4267.21	9.61	− 0.339	+ 7

Molecular weight of fully reduced form (Mw), isoelectric point (pI), and grand average of hydropathy (GRAVY). Charge was calculated from the values of Asp, − 1; Glu, − 1; Arg, + 1; Lys, + 1 under neutral conditions

Recombinant expression is an economical method of protein production. However, recombinant production of AMPs has been hindered because of their tendency to undergo degradation by host proteases and/or because they are toxic to the host cells [26, 27]. The formation of inclusion bodies is known to be a method that can prevent these undesirable events from taking place [28–31]. However, because of the high solubility of AMPs as a result of their high positive charge, simple expression often makes it difficult to form inclusion bodies of them. To solve this limitation, we developed a method in which AMPs are successfully co-expressed as inclusion bodies with aggregation-prone and negatively charged human α-lactalbumin (HLA) [32–34]. The electrostatic and hydrophobic interactions between AMPs and HLA are presumably responsible for the enhanced inclusion body formation. Using this expression system, we have previously succeeded in mass expression of Crp4, but the application of this method to other Crp isoforms has not been investigated to date.

Thus, this study examined the application of this method to the production of Crp isoforms other than Crp4. Interestingly, despite the homology between Crp isoforms, there were large differences in their expression levels. We sought to increase their expression levels by examining the causes of this difference and by investigating new methods to promote inclusion body formation. Using the various Crps ultimately obtained, we report the results of the verification of the antimicrobial activity differences between them.

## Results and discussion

### Co-expression of the various Crp isoforms with aggregation-prone proteins

**Fig. 2:**
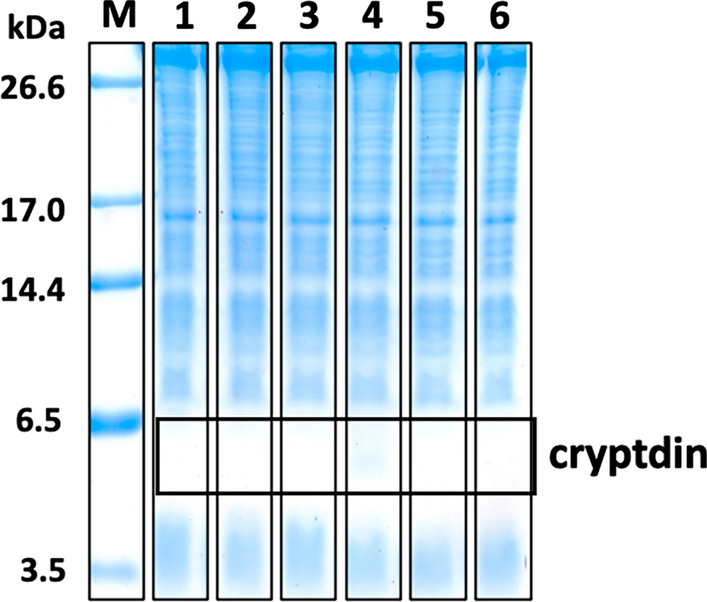
Tricine-SDS-PAGE of Crp expression without partner protein using the BL21(DE3) strain. Lane M: marker; Lanes 1–6: whole-cell lysates of cryptdins 1–6

**Fig. 3:**
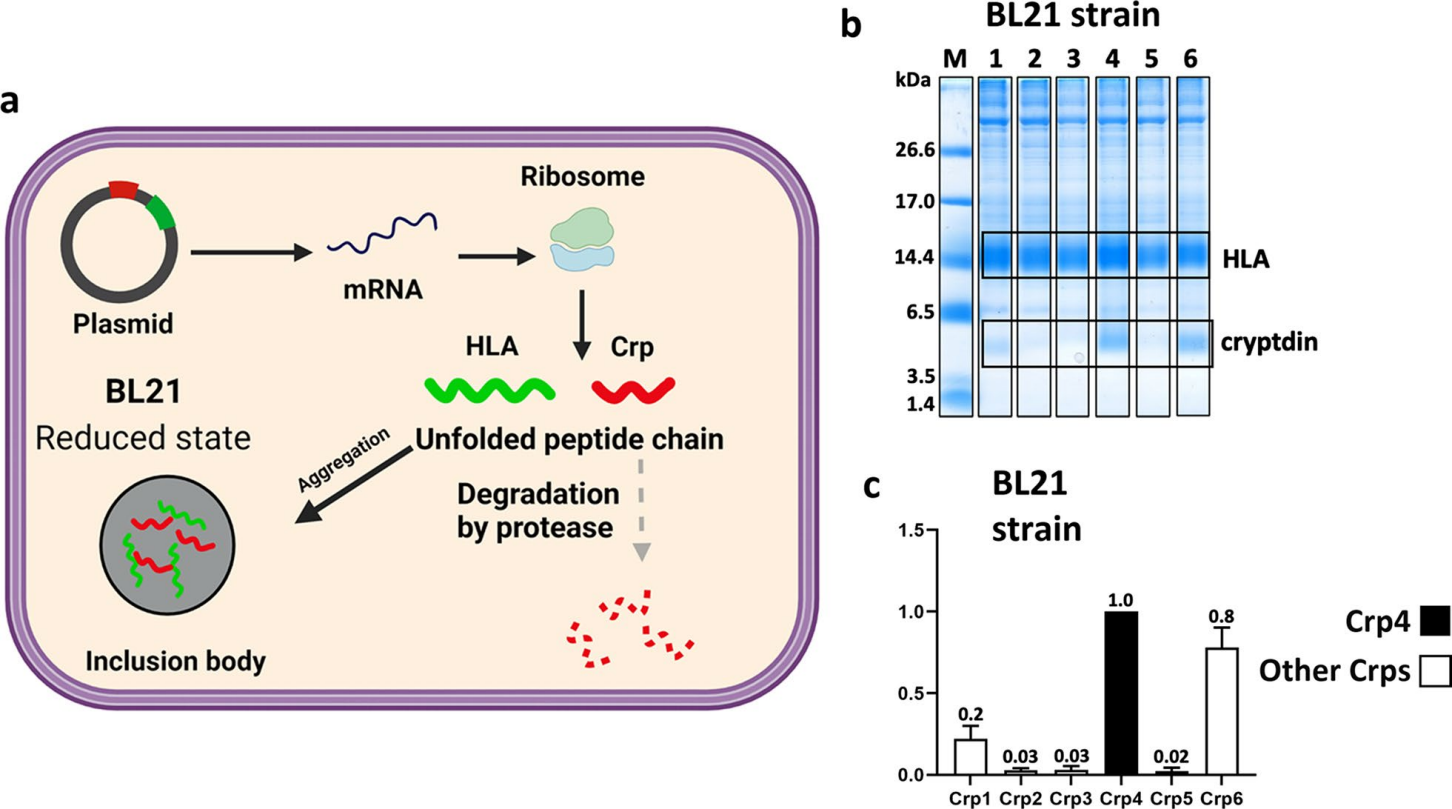
Co-expression of Crps using the *E. coli* BL21(DE3) strain. **a** Schematic outline of the method for promoting inclusion body formation using the BL21(DE3) strain. HLA, green; Crp, red. When co-expressed with aggregation-prone HLA, Crp forms a stable inclusion body (black arrow). If it does not form, host proteases degraded the expressed Crp (gray arrows). **b** Tricine-SDS-PAGE results of the expression level of Crps. Lane M: marker; Lanes 1–6: precipitates of Crp1–6. **c** The intensity data of the expression of Crps relative to Crp4. n = 3 for each

When various Crp isoforms were expressed using the pET overexpression system with *E. coli* Bl21 strain and T7 promoter-driven gene expression, little Crp expression was observed after IPTG induction (Fig. 2). It is thought that AMPs which require disulfide cross-linking to form their stable conformation are often degraded because of their instability when expressed in a reducing intracellular environment, thus resulting in a low level of production. Therefore, we tested our previously developed expression system [32–34] that promotes inclusion body formation by co-expression of AMPs with HLA, which has a high propensity for aggregation (Fig. 3a). Tricine-SDS-PAGE of overexpressed inclusion bodies showed a clear increase in Crp4 expression, consistent with our previous results, as well as an increase in Crp6 expression (Fig. 3b, c). However, although the expression was increased compared with the expression of the peptides alone, the effect was minor for Crp1 and very minor for Crp2, Crp3, and Crp5.

Two main reasons come to mind why this co-expression system did not result in enhanced expression. First, when ribosomes synthesized the different Crp isoforms, the synthesis levels were different, leading to significant differences in the final amount produced. The second possibility is that there was a difference in the efficiency of inclusion body formation, even though there was not much difference in the level of ribosome synthesis for each peptide.

### Confirmation of the amount synthesized by ribosomes using a cell-free synthesis system

**Fig. 4:**
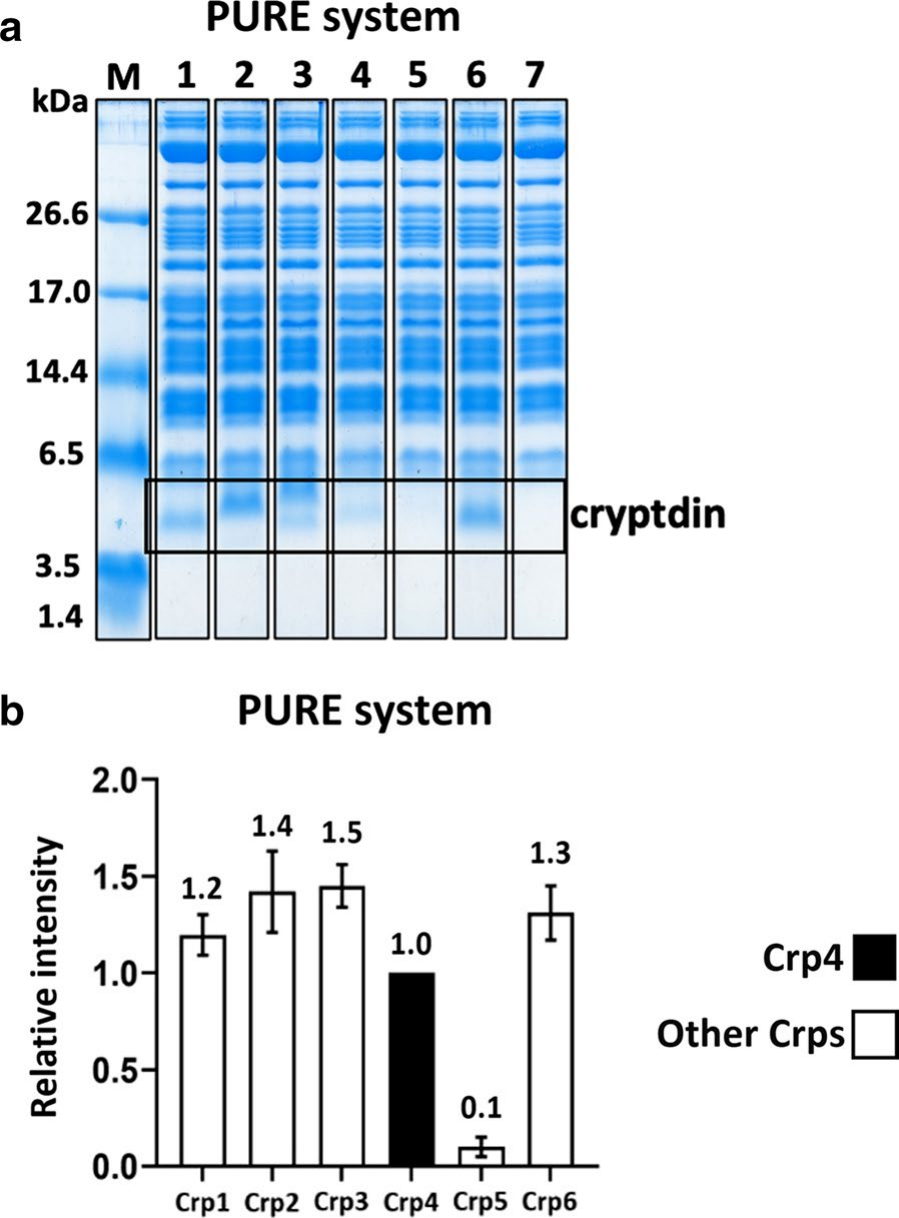
Confirmation of the synthesis of Crps by ribosomes using the PURE system. **a** Tricine-SDS-PAGE results of the synthesis of Crps. Lane M: marker; Lanes 1–6: whole-cell lysates of Crp1–6; Lane 7: negative control. **b** The intensity data of the synthesis of Crps relative to Crp4. n = 3 for each

Therefore, we first verified the amount of each Crp isoform synthesized by ribosomes using the PURE cell-free expression system [35–37]. Because the PURE system is a fully reconstituted cell-free system using *E. coli* ribosomes, it allows comparison of the synthesis of each Crp isoform by ribosomes in the absence of the effects of intracellular protease degradation [38–40]. The plasmid containing the genes coding for HLA and Crp was added to the expression system and allowed to react for 4 h, and the obtained product was confirmed by tricine-SDS-PAGE (Fig. 4). With the exception of Crp5, the expression levels of Crp1, Crp2, and Crp3 were comparable to those of Crp4 and Crp6. This suggests that the reason why low levels of insoluble granules were previously obtained with Crp1, Crp2, and Crp3 in the co-expression system with HLA in *E. coli* may be due to a limitation of the efficiency of their formation once synthesized, while for Crp5, the actual amount synthesized by the ribosomes may be limiting. As shown in Additional file 1: Table S1, Crp2, Crp3, and Crp6 have extremely high sequence homology compared with Crp1, whereas Crp5 has low homology. Therefore, the low expression of Crp5 by ribosomes compared with the other Crp isoforms may be sequence-specific. It is possible that the low production of Crp5 was not only due to the abundance of tRNA [41], but also its DNA sequence, which resulted in the structure of its mRNA not being conducive to translation by the ribosomes of *E. coli* [42]. The folding free energy of mRNA is responsible for its secondary structure formation and stability. It is known that the formation of secondary structure near the ribosome binding site, the Shine–Dalgarno (SD) sequence, of mRNA interferes with ribosome binding and reduces translation efficiency [43–45]. Thus, preventing the formation of secondary structure of such mRNAs may increase the efficiency of Crp5 synthesis by the ribosome and improve production efficiency. Silent mutations using synonymous codons that do not alter the amino acid sequence may be effective for this purpose. For example, it has been reported that by the substitution of synonymous codons, reducing the GC rate, increasing the mRNA folding free energy at the 5′-terminal ends, and thus making the secondary structure of mRNA more unstable, can lead to an increase in protein expression [46, 47]. In the production of Crp5, it may be possible to increase production efficiency by considering using such techniques to increase the amount of Crp5 synthesized on the ribosome. In contrast, it is unclear why Crp1, Crp2, and Crp3, which are highly homologous to Crp6 and are not limited by ribosomal synthesis, were not efficient in terms of inclusion body formation. Previous studies have shown that electrostatic and hydrophobic interactions play an important role in efficient inclusion body formation in our co-expression system. However, we could not find any trend in Crp family isoelectric points or GRAVY scores (Table 1) that might explain the success or failure of inclusion body formation. Various culture conditions, such as the type of medium, the culture temperature, and IPTG induction conditions were examined with the aim of achieving more efficient inclusion body formation, but unfortunately, no improvement in the expression was observed (data not shown).

### Enhancement of inclusion body formation by disulfide cross-link formation

**Fig. 5:**
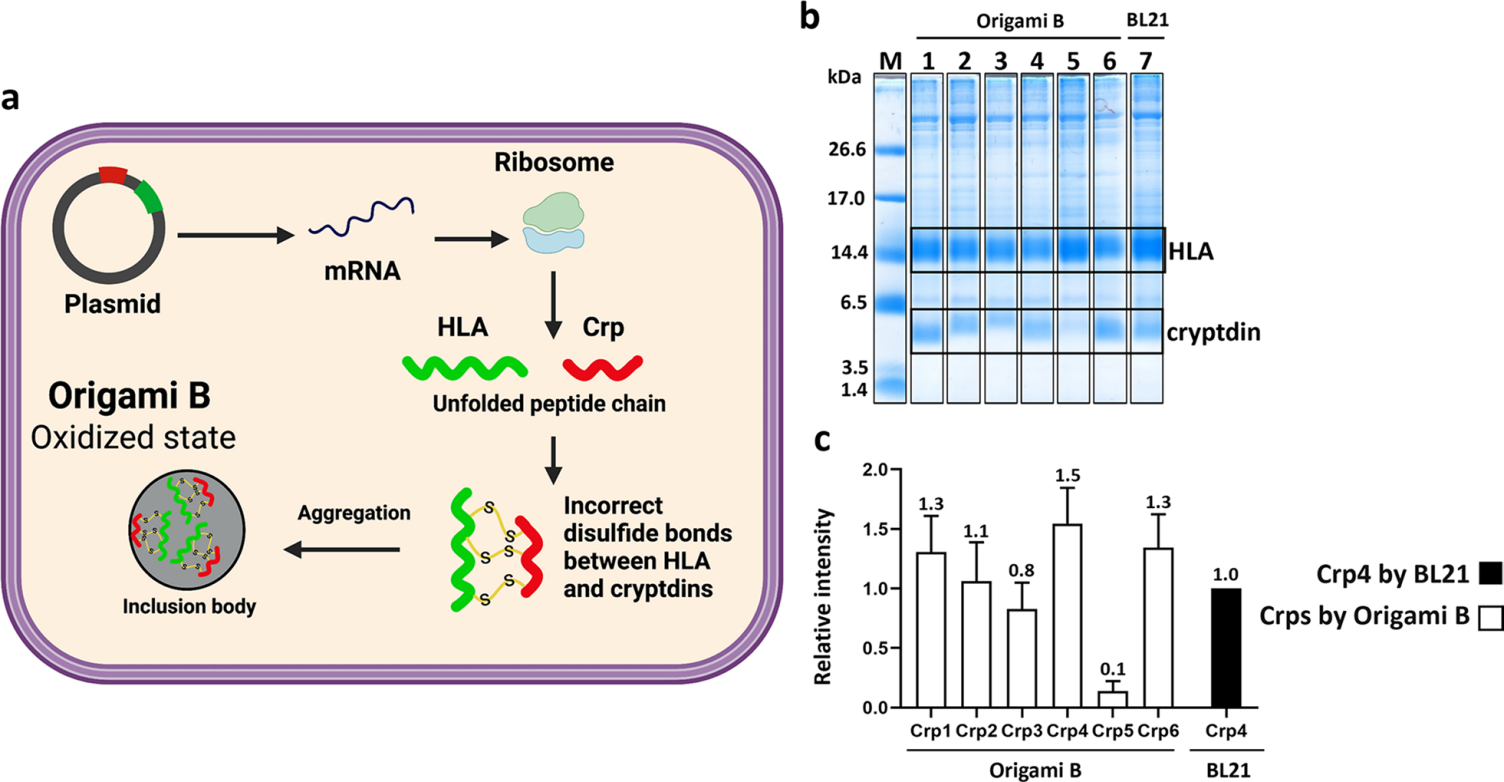
Co-expression of Crps using the *E. coli* Origami™ B (DE3) strain. **a** Schematic outline of the newly investigated method for promoting inclusion body formation by disulfide bond cross-linking using the *E. coli* Origami™ B (DE3) strain. HLA, green; Crp, red; disulfide cross-link, yellow. Use of the Origami™ B (DE3) strain, which has an intracellular oxidative environment, as an expression host allows the formation of non-natural disulfide cross-links between aggregation-prone HLA and Crp. This is expected to result in more efficient inclusion body formation. **b** Tricine-SDS-PAGE results of the expression of Crps. Lane M: marker; Lanes 1–6: precipitates of Crp1–6. Lane7: Precipitate of Crp4 by BL21 strain was used as a control. **c** The intensity data of the expression of Crps relative to Crp4 using the BL21(DE3) strain. n = 3 for each

Based on these results, we sought to promote the formation of insoluble granules by forming non-natural disulfide cross-links between the partner protein and the Crps (Fig. 5a). The cellular redox state of normal *E. coli*, such as BL21, is reducing, which generally precludes disulfide cross-link formation. In contrast, the Origami™ B strain is an expression host developed to obtain proteins with natural disulfide bond formation in the cell by the introduction of mutations in *trxB* and *gor* [48, 49], thereby creating an oxidizing redox state in the cells. Therefore, in this study, we sought to promote the formation of non-natural disulfide cross-links, contrary to the original purpose of using the Origami™ strain. As expected, using the Origami™ B strain greatly improved the expression levels of Crp1, Crp2, and Crp3 (Fig. 5b, c). There was not, however, a significant increase in Crp5 expression, suggesting that, as expected, the amount of synthesis by the ribosomes is limiting for Crp5.

The Origami™ strain is a frequently used host for the efficient formation of wild-type disulfide bonds and has been used with great success [50–52]. However, to the best of our knowledge, this is the first report of the use of unnatural-type disulfide bond formation in cells to promote inclusion body formation. This approach has great potential as a versatile technique for the efficient formation of inclusion bodies of peptides that are otherwise prone to degradation in the cell.

### Large-scale overexpression and purification of Crps

Large-scale culture and purification conditions were investigated to obtain abundant quantities of individual Crp peptide isoforms. After assessing various incubation conditions, the optimal induction condition was found to be 30 °C for 6 h [53, 54]. The inclusion complexes containing Crp and HLA were separated by solubilizing them with a denaturing agent containing a reducing agent to cleave the disulfide bonds between them, followed by purification using cation exchange chromatography (Additional file 1: Fig. S1). Tricine-SDS-PAGE showed that each main peak contained a Crp isoform, but the amount of Crp5 was insufficient to allow further experiments to be conducted (data not shown). One method for forming an inclusion body of the peptide is to fuse the peptide to a highly insoluble protein, such as KSI, as used with the pET31 expression vector [55, 56]. However, this method requires chemical cleavage with CNBr, or another chemical cleavage must be performed, to separate the peptide from the fusion protein. Such cleavage methods may result in poor selectivity and undesirable side reactions. Our method using co-expression is superior in the sense that it can efficiently separate the partner HLA from the target peptide using a simple reducing agent containing a denaturing agent.

**Fig. 6:**
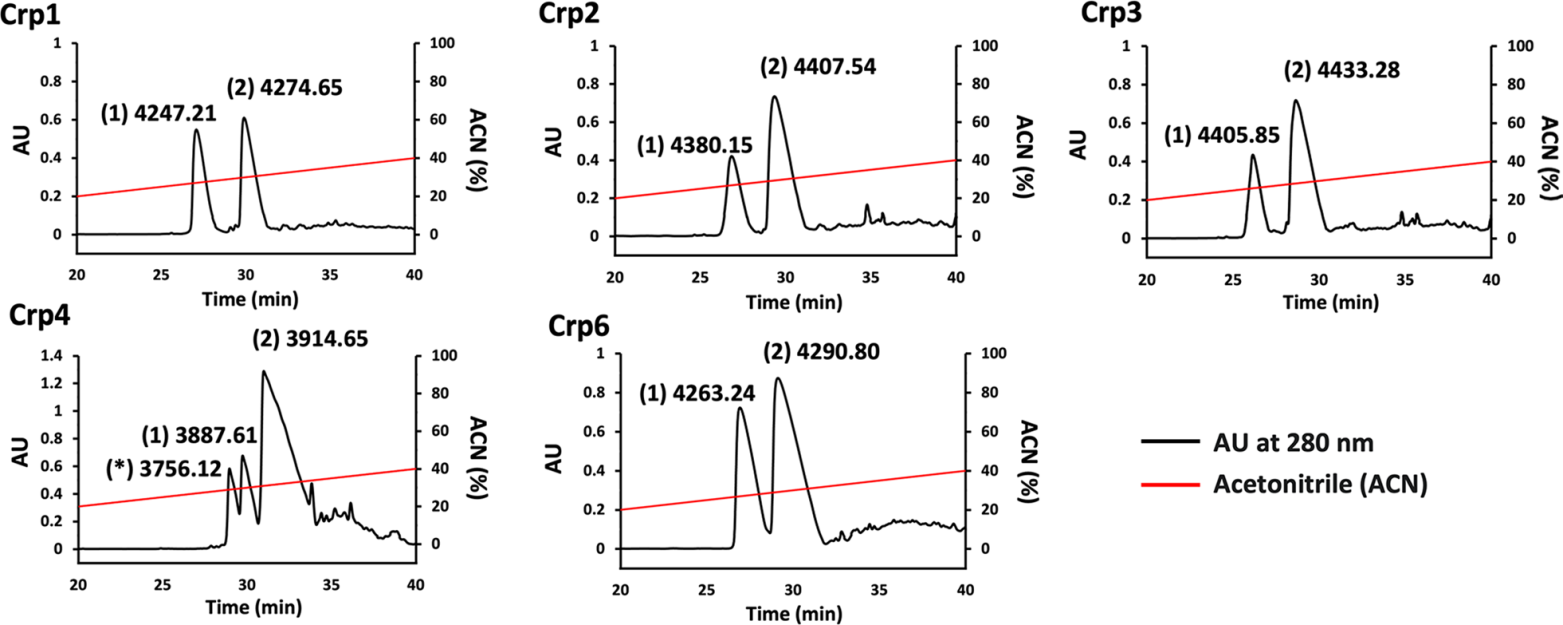
Large-scale purification of refolded Crps by RP-HPLC. The molecular weight of each peak was determined by MALDI-TOF mass spectrometry. For Crp1, Crp2, Crp3, and Crp6, the two observable peaks are: (1) Crps; and (2) formyl Crps (due to the difference of approximately 28 Da determined by mass). For Crp4, the three observable peaks are: (*) Crp4 without methionine (due to the side chain length of the second amino acid of Crp4 [59]); (1) Crp4; and (2) formyl Crp4. Peptides produced in 500 mL of medium were loaded

Subsequently, refolding was performed by removal of the urea, β-mercaptoethanol, and salts by dialysis. With the exception of Crp5, the product obtained after dialysis could be purified by reverse-phase HPLC. Each purified Crp isoform could thereby be separated into two or three peaks, as shown in Fig. 6. The products were analyzed using MALDI-TOF mass spectrometry, and the results obtained are indicated above the peaks in Fig. 6. Among these, the molecular weight of peak (1) of each Crp isoform was apploxymetly 6 Da smaller than that of its fully reduced peptide (Table 1), indicating that Crps that refolded successfully by forming three disulfide bonds were obtained using this expression purification system. In addition, peak (2) had a molecular weight 28 Da larger than that of peak (1), which is thought to be due to modification of the N-terminal methionine by a formyl group [57, 58]. For Crp4, a peak (*) with a smaller molecular weight was observed earlier than peak (1). This is because the side chain length of glycine, which is the amino acid following the N-terminal methionine of Crp4, is sufficiently short for the N-terminal methionine to be cleaved by methionine aminopeptidase [59]. The yields of the various products are listed in Table 2.

**Table 2:** The yield of each Crp isoform

Purification step		Yield (mg/L of culture)				
		Crp1	Crp2	Crp3	Crp4	Crp6
CIEX	Crude extract	5.0	5.2	5.3	8.5	9.1
RP-HPLC	Crps	1.5	0.95	0.92	1.9^a^	1.8
	Formyl-Crps	1.7	2.3	2.3	5.3	3.0
Deformylation	Crps	1.4	2.0	1.9	4.2	2.2
Total Crps		2.9	3.0	2.8	6.1^a^	4.0

The total yield of Crps was obtained by adding the amount of Crps after purification by RP-HPLC to the amount of Crps after deformylation

^a^Crp4 represents the sum of the total amounts of Crp4 with and without methionine

The residual formyl groups on the N-terminal methionine were further investigated. For comparison, the results of Crp6 expression using the BL21 strain showed that the product also contained Crp6 with N-terminal methionine formylation (Additional file 1: Fig. S2); however, the amount was clearly lower than that obtained using the Origami™ B strain. Normally, the N-terminal formyl group is removed by the intracellular peptide deformylase (PDF) [58, 60]. However, it has been reported that when excessive amounts of recombinant proteins are expressed, the degradation efficiency of PDF is reduced, and the products contain proteins with formylmethionine [57]. Furthermore, it has been reported that nascent peptides with less than 50 amino acids readily retain the formyl group [61]. Also, the activity of PDF is affected by certain metal ions in *E. coli*, such as Fe^2+^ [62–64]. The present results suggest that the oxidative intracellular environment of the Origami™ B strain may have affected the Fe^2+^ concentration, decreased PDF activity, and hence resulted in an increase in the content of formylated Crp in the product.

### Attempts to increase the yield by chemical deformylation

**Fig. 7:**
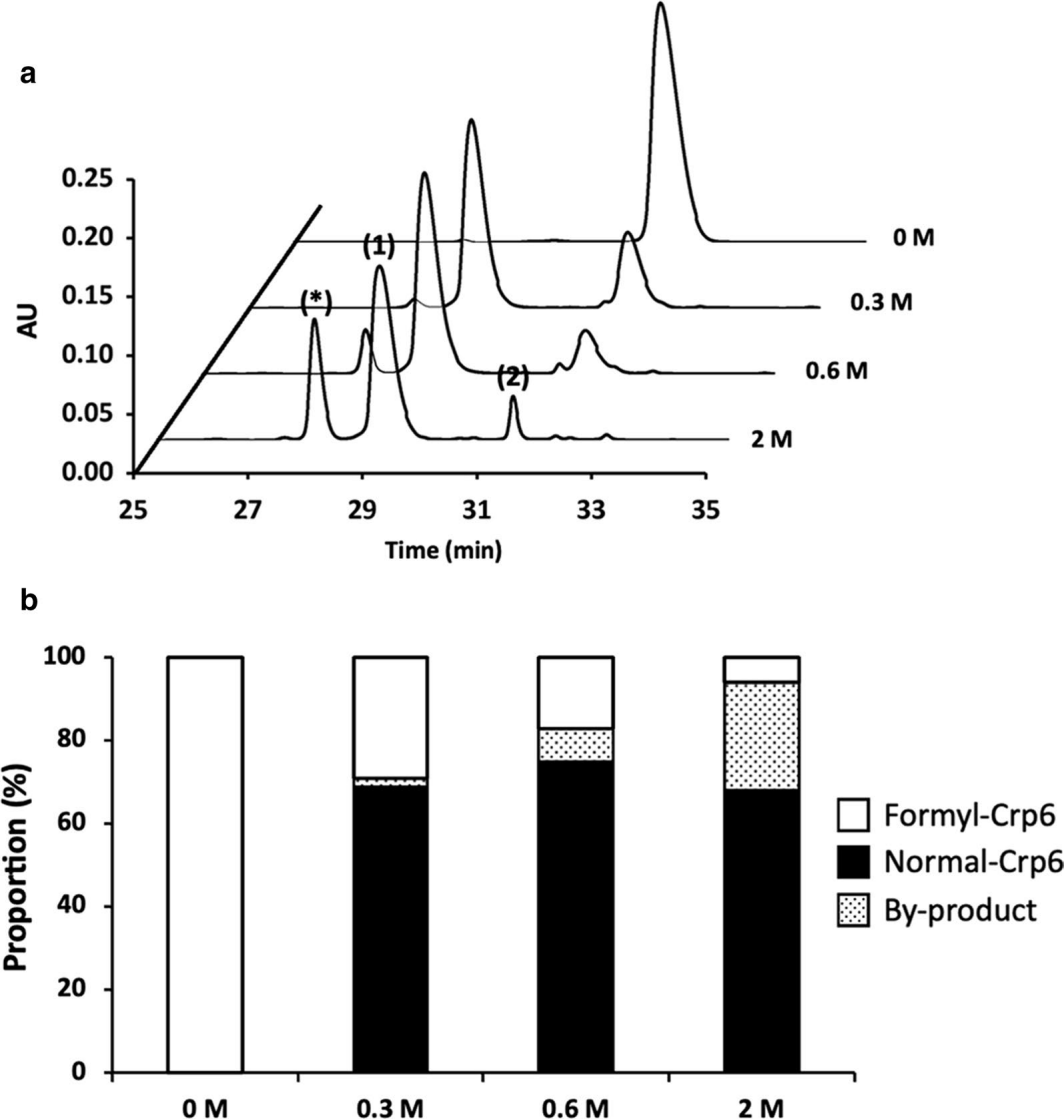
The result of deformylation of Crp6 by acid hydrolysis. **a** RP- HPLC results of Crp6 treated with different concentrations of HCl, 0 (control), 0.3, 0.6, and 2 M HCl. The three observable peaks are: (*) by-product; (1); Crp6 after deformylation; and (2) undeformylated Crp6. **b** Bar graph showing the proportion of each product, confirmed by the peak area by RP-HPLC. The molecular weight of Crp6 was determined by MALDI-TOF mass spectrometry. Approximately 100 µg of Crp6 was loaded

To further increase the yield of Crps, we first investigated the conditions for deformylation by the acidic hydrolysis method [65, 66] using purified formylated Crp6. The results of the analysis using RP-HPLC after reaction with various concentrations of HCl for more than 20 h are shown in Fig. 7a. After acid hydrolysis, three main peaks were observed. The amount of Crp6 that was successfully deformylated (Peak 1) from the formylated Crp6 (Peak 2) increased in an HCl concentration-dependent manner, but the byproducts (peaks indicated by *), which were further hydrolyzed between the aspartic acid at the fourth position and the leucine at the fifth position, also increased. Among these, more than 70% of the formyl-Crp6 was successfully deformylated into Crp6 after exposure to 0.6 M hydrochloric acid, and the proportion of byproducts was not high (Fig. 7b). Therefore, considering the proportion of deformylation and byproducts, hydrolysis with 0.6 M HCl was determined to be the optimal condition, and other Crps were also treated with 0.6 M HCl (Additional file 1: Fig. S3), and their component ratios are listed in Additional file 1: Table S2. The proportion of successful deformylation for each Crp isoform exceeded 80%.

The yields of successfully deformylated Crps and the final Crp isoform yields obtained were calculated and are presented in Table 2. Chemical deformylation resulted in a significant increase in the production of each Crp isoform, with yields reaching at least 3 mg/L of medium.

### Comparison of the characteristics of the antimicrobial activity of Crps

**Fig. 8:**
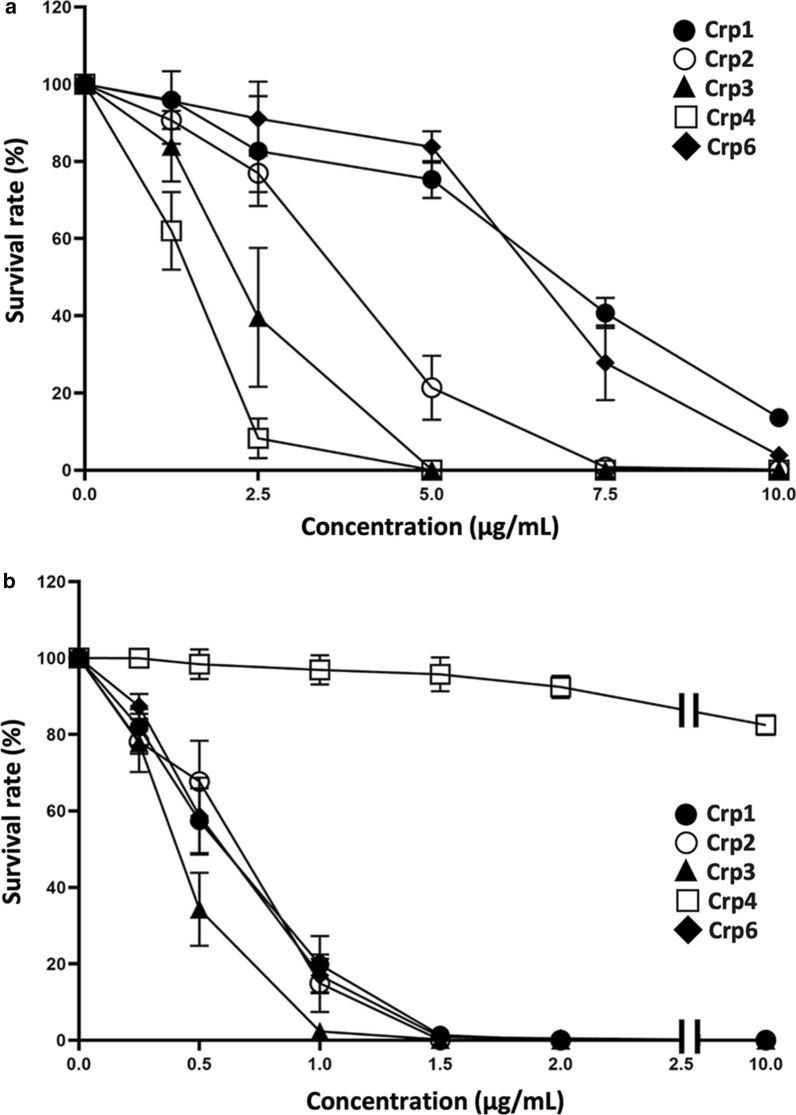
Antimicrobial activity of Crps against bacteria. **a** Approximately 1 × 10^7^ CFU/mL *E. coli* was exposed to peptides at 0, 1.25, 2.5, 5, 7.5, and 10 µg/mL; **b** approximately 1 × 10^7^ CFU/mL *S. aureus* was exposed to peptides at 0, 0.25, 0.5, 1, 1.5, 2, and 10 µg/mL. Data are presented as means ± the standard error of the mean (SEM). n = 6 for both **a** and **b**

The bactericidal activity of Crps against Gram-positive bacteria (*Staphylococcus aureus*) and Gram-negative bacteria (*Escherichia coli*) was analyzed. Crp4 exhibited the strongest antimicrobial activity against *E. coli*, followed by Crp3 and Crp2. Crp1 and Crp6 exhibited the weakest activities (Fig. 8a). For instance, 5.0 µg/mL Crp3 or Crp4 was sufficient to kill all *E. coli*, but the same concentration of Crp1 or Crp6 could only kill 20% of the bacteria. However, Crp4 exhibited very low activity against *S. aureus* (Fig. 8b). At a concentration of 10 µg/mL, it still only killed approximately 10% of the bacteria. In contrast, the other four Crps all exhibited very strong bactericidal activity, with their minimum bactericidal concentration (MBC) values below 1.5 µg/mL, which was much lower than the MBC of these Crps against *E. coli*. These trends clearly indicate that each Crp isoform has a very different antimicrobial spectrum.

The number and variety of bacteria in different parts of the mouse small intestine vary. Paneth cells at the bottom of small intestinal crypts of the mouse respond immediately to stimulation by a variety of pathogens, including many different bacteria, to secrete intracellular granules rich in Crps [67–69], killing pathogenic bacteria to contribute to innate immunity. It has been reported that different parts of the mouse small intestine also express each Crp isoform to different degrees [13, 70, 71], suggesting that each Crp isoform may play a different role in the defense of the small intestine [25]. For this reason, our finding that each Crp isoform exhibited a different antimicrobial spectrum is very interesting. To the best of our knowledge, no reports to date have directly or simultaneously compared the MBCs of many types of Crps. In the case of *E. coli* in this study, with the exception of Crp4, the other four Crps exhibited activities that were highly consistent with their electric charge (Table 1), indicating that in the case of *E. coli*, the activity exhibited by Crps is likely to be affected by the charge strength. The microbicidal activity of these four Crps (Crp1, Crp2, Crp3, and Crp6), but not Crp4, against *E. coli* was stronger the higher their positive charge (Table 1). These four Crp isoforms have a high degree of sequence homology, differing by only 2–3 amino acids (Fig. 1, Additional file 1: Table S1). This suggests that the activity of these Crps against *E. coli* depends on the strength of their electrostatic interaction with the membrane and that the basic mechanism of action of membrane disruption is similar. Unlike *E. coli*, the activity of Crps against *S. aureus* was not specifically related to their electric charge and differed largely between Crp4 and the other Crps. This particularly weak activity against Gram-positive bacteria may be because Crp4 has a sequence that is considerably different from the other Crps, and the mechanism of action is also different. Additionally, the antibacterial activity of Crp3 against *S. aureus* was slightly stronger than that of Crp1, Crp2, and Crp6. The only primary sequence difference between Crp3 and Crp2 is at position 11 (Crp3 has a lysine and Crp2 has a threonine). It has been reported that amino acids at positions 11 and 16 of Crp1, Crp2, Crp3, and Crp6 are predicted to be located at conserved turns on the molecular surface based on analogy with HNP-1, HNP-3, NP-2, and NP-5 [19, 24, 72]. Based on these findings, the amino acid at position 11 may affect the interaction between Crps (Crp1, Crp2, Crp3, and Crp6) and *S. aureus.*

### Comparison of steric structures by circular dichroism (CD)

**Fig. 9:**
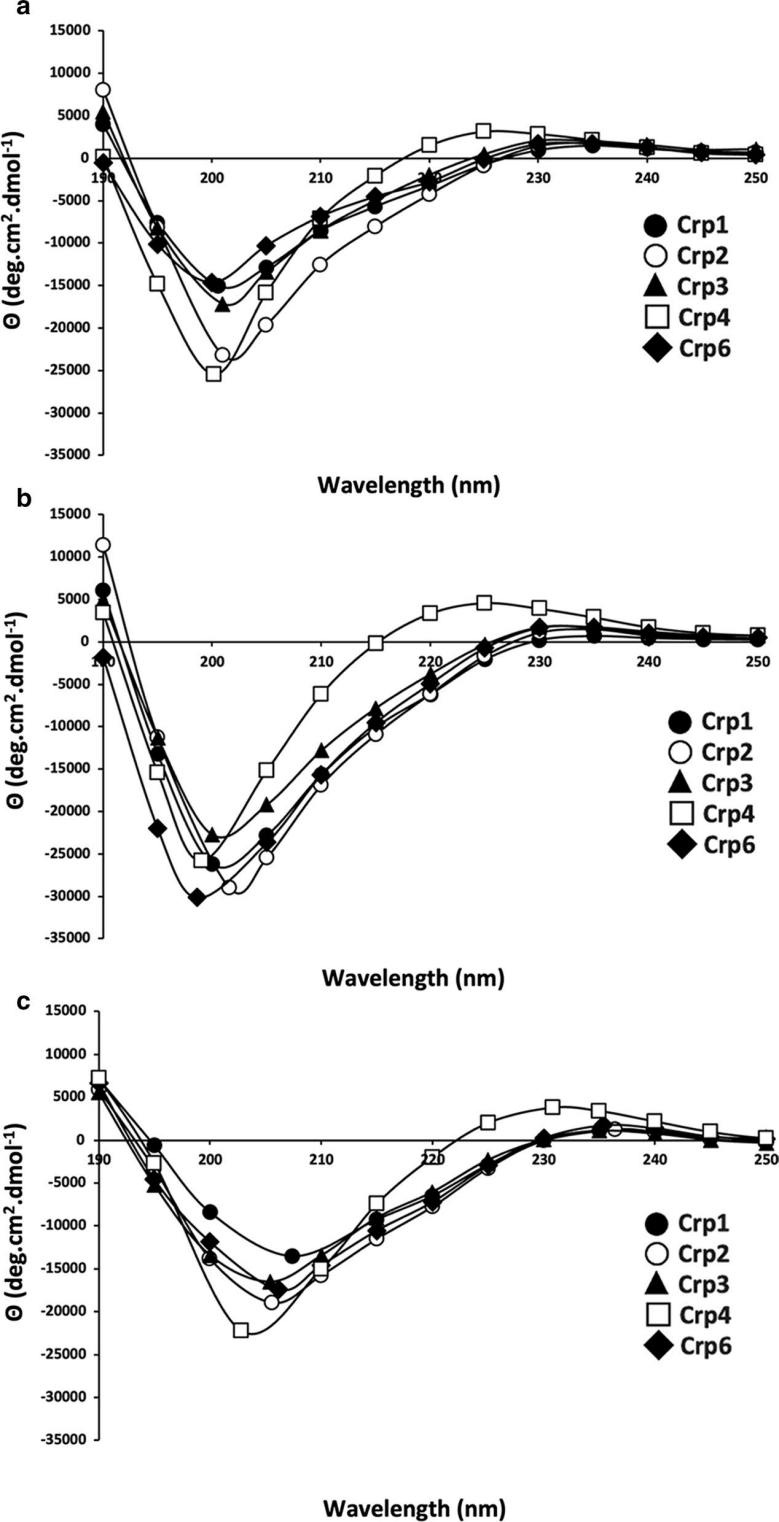
Circular dichroism (CD) spectra measured from 280 to 190 nm at 25 °C. Each peptide, at 30 µM, was measured in **a** 10 mM phosphate-buffered saline (PBS, pH 7.4); **b** 40% trifluoroethanol (TFE); **c** 10 mM sodium dodecyl sulfate (SDS)

To obtain information on the steric structures of the five Crps, CD spectra were measured under different conditions, and the results are shown in Fig. 9. The steric structure of Crp4 has been studied previously using CD and NMR [20, 73, 74], and it has been reported that the three disulfide bonds stabilize the three β-strands. Consistent with previous results, Crp4 in aqueous solution exhibited a negative maximum at approximately 200 nm and a broad positive maximum at approximately 225 nm (Fig. 9a). These spectral features were generally common in other Crps, suggesting that they form a conformation similar to that of Crp4. Furthermore, CD spectra were measured in highly hydrophobic (40% TFE) and membrane-mimetic (10 mM SDS) environments, but no significant changes in the spectra in aqueous solution were observed for any of the Crps (Fig. 9b, c). This suggests that all of the Crps had a stable secondary structure due to disulfide cross-linking and that their steric structures were stable in various environments. Under all measurement conditions, the CD spectrum of Crp4 exhibited a stronger broad peak at 225 nm compared to the other Crps. Unfortunately, it is unclear at this time whether this reflects a difference in the steric structure associated with the antimicrobial spectrum characteristic of Crp4 or simply a difference in the primary sequence.

## Conclusion

In this study, to form inclusion bodies so as to avoid degradation of cysteine-containing antimicrobial peptides, we devised a method involving the use of *E. coli* with an oxidative intracellular environment and applied it to the production of the Crp family of peptides. This approach allowed successful production of five recombinant Crp isoforms. Crp5, which could not be produced, was found to be limited by a low level of synthesis at the ribosomal level. Although *E. coli* strains with an oxidative intracellular environment produced peptides with a formyl group on the N-terminal methionine, we showed that deformylation by hydrolysis under acidic conditions was effective at increasing production. The Crp family of peptides obtained using this experimental system exhibited significant differences in their antimicrobial spectra, even though their basic steric structures appeared to be similar. The expression system developed in this study is expected to elucidate the mechanism of action responsible for differences in the antimicrobial spectrum of the Crp family of peptides. In particular, the production of peptides labeled with ^15^N and ^13^C stable isotopes using this expression system is expected to be useful for future detailed structure and interaction analyses by NMR.

## Materials and methods

### Strains, vectors, and reagents

DNA cloning was performed using the *E. coli* DH5α strain and pCOLADuet1 and pET-16b plasmid. *E. coli* BL21(DE3) and *E. coli* Origami™ B(DE3) were used for expression analysis. All *E. coli* strains and vectors were obtained from Novagen. DNA extractions were performed with a FastGene^®^ Plasmid Mini Kit (NIPPON Genetics Co., Ltd.), and the FastDigest^®^ restriction enzymes XhoI, NdeI, NcoI, BamHI, and BglII were purchased from Thermo Fisher Scientific.

### Plasmid construction

**Fig. 10:**
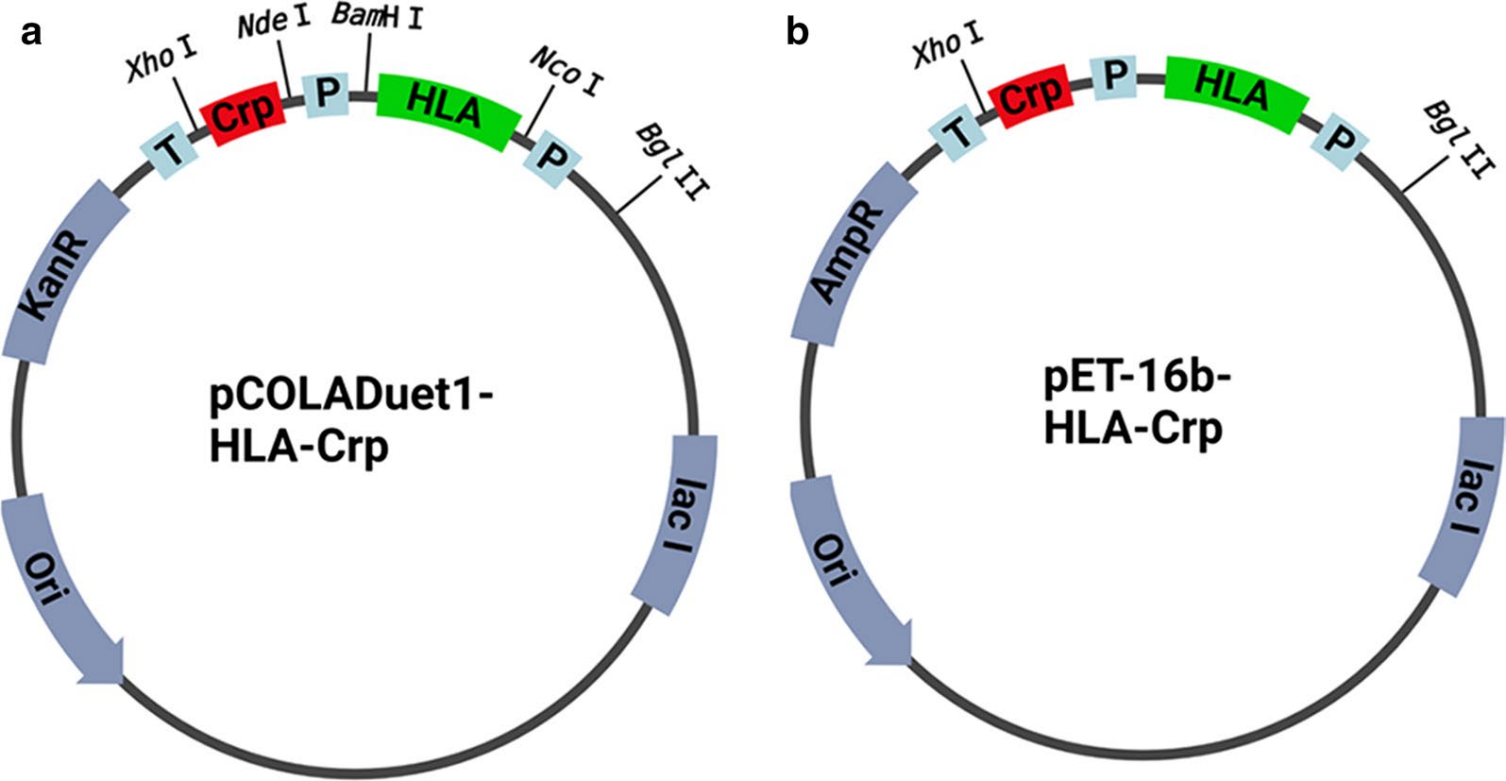
Schematic representation of the expression vectors. **a** pCOLA-Duet1-HLA-Crp vector. **b** pET16b-HLA-Crp vector. P, T7 promoter; HLA, HLA gene; Crp, Crp gene; T, T7 terminator

In this study, because co-expression was required, the DNA sequences of the partner protein HLA and the target Crps were inserted into the pCOLADute1 plasmid (Fig. 10a). In addition to the pCOLADuet vector, which is kanamycin-resistant and has a ColA replication origin, similar insertions were made into the pET-16b plasmid, which is ampicillin-resistant and has a ColE1 replication origin (Fig. 10b). Thus, the vectors containing the HLA and Crps genes can be expressed in the kanamycin-resistant Origami™ B strain. The 372 bp fragment encoding the partner protein HLA was inserted into the NcoI and BamHI restriction sites of the vector pCOLA-Duet1, and the 99–108 bp fragments encoding the target protein Crp isoforms were inserted into the NdeI and XhoI restriction sites. *E. coli* DH5α were transformed with the plasmids, and positive transformants were screened on lysogeny broth (LB) plates and confirmed using a DNA sequencer (Applied Biosystems 3130 Genetic Analyzer). The resulting pCOLA-Duet1 plasmids were digested with the endonucleases BglII and XhoI, and the fragments were inserted into pET-16b. After confirmation by DNA sequencing, the expression strains were transformed with the vectors.

### Expression of Crps in the *E. coli* expression system

The *E. coli* BL21(DE3) and Origami™ B(DE3) strains transformed with the plasmid pET-16b-HLA-Crps were inoculated into 5 mL of LB medium containing 50 µg/mL ampicillin and incubated at 37 °C with shaking at 180 rpm until the absorbance at 600 nm reached 1.0. Expression was induced by the addition of IPTG, and the bacterial liquid was subjected to shaking at 180 rpm for 4 h. The cells were collected by centrifugation at 10,000 rpm for 3 min and washed with disruption buffer (20 mM Tris-HCl (pH 8) and 1 mM EDTA). One milliliter of the medium was resuspended in 100 µL of buffer and disrupted with a sonicator (Misonix™ Microson™ Ultrasonic Cell Disruptor XL2000) on ice. The mixture was centrifuged at 10,000 rpm for 3 min. The amount of protein in the obtained supernatant and pellet was confirmed by Tricine-SDS-PAGE (n = 3 for each).

### Expression in PURE system

The plasmid pET-16b-HLA-Crps was added to the PURE system (PUREfrex2.0) at a concentration of 2 ng/µL. The entire reaction was carried out in an RNase-free environment. The cells were incubated at 37 °C for 4 h. The amount of protein in the reaction system was confirmed by Tricine-SDS-PAGE (n = 3 for each).

### Large-scale expression of Crps for purification

Origami™ B transformed with plasmid pET-16b-HLA-Crps was inoculated into 50 mL of LB medium containing 50 µg/mL ampicillin and incubated overnight at 30 °C and 180 rpm. The mixture was centrifuged at 6000 rpm for 5 min and resuspended in 500 mL of LB medium containing 50 µg/mL ampicillin. The culture was incubated at 37 °C with shaking at 120 rpm. Once the absorbance at 600 nm reached 1.0, 1 mM IPTG was added to induce protein expression. After culturing at 30 °C with shaking at 120 rpm for 6 h, the bacterial solution was centrifuged at 6000 rpm for 10 min, and the bacterial cells were washed with disruption buffer and stored.

### Purification and analysis of Crps

The obtained bacterial cells were resuspended in fragmentation buffer [20mM Tris-HCl (pH8.0), 1 mM EDTA], crushed in an ultrasonic crusher (Insonator 201 M, KUBOTA) at 180 W for 30 min, and centrifuged at 4300×*g* for 20 min to obtain a precipitate containing inclusion bodies. The pellet was resuspended in a solubilization buffer [50 mM glycine-NaOH (pH8.5), 3 mM EDTA, and 6 M urea, final pH 9.0] and incubated at 24 °C with shaking at 180 rpm for 1 h so that almost all of the inclusion bodies were dissolved. After centrifugation at 7000×*g* for 20 min, the supernatant was loaded onto an SP Sepharose® FAST FLOW cation exchange column (Cytiva™) equilibrated with 50 mM glycine-NaOH (pH 8.5), 3 mM EDTA, 6 M urea, and 20 mM β-mercaptoethanol, final pH 9.0. The bound Crps were eluted using a linear gradient of 0–1 M NaCl buffer. The fractions containing Crps were refolded twice at 4 °C using refolding buffer containing 50 mM glycine-NaOH (pH8.5), 2 M urea, 3 mM reduced glutathione, 0.3 mM oxidized glutathione, and 10% glycerol, final pH 9.0, for approximately 12 h each time, after which 0.1% acetic acid was dialyzed overnight to remove other compounds in the system. The dialyzed Crps were purified by RP-HPLC using a COSMOSIL® Protein-R column (Nacalai Tesque). Elution was performed using a linear gradient of 0–50% acetonitrile and 0.1% TFA. Yields were calculated based on the absorbance at 280 nm. The molecular weights of the eluted Crps were determined by MALDI-TOF-MS (autoflex™ speed, Bruker).

### Deformylation

We added 0, 7.5, 15, and 50 µL of 6 M hydrochloric acid to 100 µL of formyl-Crp6 at a concentration of approximately 1 mg/mL, and ddH_2_O was added to bring the total volume to 150 µL. The final concentration of hydrochloric acid in each group was 0, 0.3, 0.6, and 2 M. A 100 µL aliquot of each of the other formyl-Crps at a concentration of approximately 1 mg/mL was deamidated with 0.6 M hydrochloric acid.

After incubation at 37 °C for more than 20 h, the reaction was terminated by the addition of 850 µL ddH_2_O, the pH was adjusted to 2–3 using NaOH, and RP-HPLC was used to analyze the amount of each component.

### Antimicrobial activity assay

Gram-positive bacteria (*Staphylococcus aureus*, ATCC6538p) and Gram-negative bacteria (*Escherichia coli*, ATCC43827) were used to test the antibacterial activity. Both of these bacterial strains were cultured in 3% TSB medium. After shaking at 37 °C until the absorbance at 600 nm was 0.4, the cultures were centrifuged at 9300×*g* for 5 min, and the pellets were washed twice with 10 mM PBS, (pH 7.4), resuspended, and diluted tenfold (*E. coli*) and 20-fold with PBS. (*S. aureus*). After the Crps were freeze-dried, they were diluted to the corresponding concentrations with PBS. The diluted bacteria (20 µL aliquots) were mixed with 20 µL of the diluted Crps, and the concentration of the bacteria was 1 × 10^7^ CFU/mL. The mixtures were then incubated at 37 °C for 1 h, diluted 1000 times, and 50 µL was added to solid medium containing 3% TSB. After overnight culturing at 37 °C, the survival rate was calculated by colony counting (n = 6 for each).

### Circular dichroism (CD) measurements

Circular dichroism (CD) data were collected using a Jasco J 725 spectropolarimeter (Jasco Inc.). Spectra were collected from 250 to 190 nm and scanned at 20 nm/min at 25 °C. The bandwidth was 1.0 nm, and each data point was scanned four times under nitrogen gas. The spectra were measured in three different environments: 10 mM phosphate-buffered saline (PBS), pH 7.4; 40% trifluoroethanol (TFE); and 10 mM sodium dodecyl sulfate (SDS).

The mean residue ellipticity values, θ, were calculated using the formula below:

1 $$\theta =\frac{\theta_{observed}}{10\times n\times C\times l}$$

where n is the number of amino acid residues, C is the peptide concentration, and l is the optical pass length of the cell.

## Supplementary Information

**Additional file 1: Table S1.** Amino acid sequence identity and similarity of Crps. **Table S2.** Quantitative results of the amount of deformylation of Crps. **Figure S1.** Cation exchange chromatography (CIEX) results for Crps. (a) Crp1; (b) Crp2; (c) Crp3; (d) Crp4;(e) Crp5; and (f) Crp6. **Figure S2.** RP-HPLC results after purification of Crp6 using the BL21 strain. The two observable peaks are: (1) Crp6; and (2) formyl Crp6. The result for Crp6 by using the Origami™ B strain (Fig. 3e). The proportion of mature Crp6 was higher. Peptides produced in 1 L of medium were loaded. **FigureS3.** RP-HPLC results for Crp deformylation. The figure shows the results for Crps treated with 0 M (control) and 0.6 M HCl. The two observable peaks are: (1) Crps after deformylation; and (2) undeformylated Crps. The molecular weight of the mature Crps was determinedusing MALDI-TOF mass spectrometry. Approximately 100 μg of Crps were loaded.

## Data Availability

All data generated or analyzed in this study are included in this published article. The datasets used and/or analyzed in the current study are available from the corresponding author upon reasonable request.

## Abbreviations

AMPs: Antimicrobial peptides
Crp: Cryptdin
HLA: Human α-lactalbumin
Mw: Molecular weight
pI: Isoelectric point
GRAVY: Grand average of hydropathy
RP-HPLC: Reverse-phase high-performance liquid chromatography
MBC: Minimum bactericidal concentration
OD: Optical density
PBS: Phosphate-buffered saline
TFE: Trifluoroethanol
SDS: Sodium dodecyl sulfate
CD: Circular dichroism
MALDI-TOF-MS: Matrix-assisted laser desorption/ionization-time of flight mass spectrometry
SEM: Standard error of the mean
CFU: Colony-forming unit
