# Supplementary material for: Efficient recombinant production of mouse-derived cryptdin family peptides by a novel facilitation strategy for inclusion body formation
Source: Microb Cell Fact. 2023 Jan 13;22:9. doi: 10.1186/s12934-023-02016-2 (PMC9838031; doi:10.1186/s12934-023-02016-2)
Supplement: Supplementary file 1 — Additional file 1: Table S1. Amino acid sequence identity and similarity of Crps. Table S2. Quantitative results of the amount of deformylation of Crps. Figure S1. Cation exchange chromatography (CIEX) results for Crps. (a) Crp1; (b) Crp2; (c) Crp3; (d) Crp4;(e) Crp5; and (f) Crp6. Figure S2. RP-HPLC results after purification of Crp6 using the BL21 strain. The two observable peaks are: (1) Crp6; and (2) formyl Crp6. The result for Crp6 by using the Origami™ B strain (Fig. 3e). The proportion of mature Crp6 was higher. Peptides produced in 1 L of medium were loaded. FigureS3. RP-HPLC results for Crp deformylation. The figure shows the results for Crps treated with 0 M (control) and 0.6 M HCl. The two observable peaks are: (1) Crps after deformylation; and (2) undeformylated Crps. The molecular weight of the mature Crps was determinedusing MALDI-TOF mass spectrometry. Approximately 100 μg of Crps were loaded. [file 12934_2023_2016_MOESM1_ESM.docx]

**Additional file 1: Table s1.** Amino acid sequence identity and similarity of Crps.

| Identity and similarity (%) | Crp1 | Crp2 | Crp3 | Crp4 | Crp5 | Crp6 |
| --- | --- | --- | --- | --- | --- | --- |
| Crp1 |  | 91.67 | 91.67 | 39.47 | 55.56 | 94.44 |
| Crp2 | 94.44 |  | 97.22 | 42.11 | 58.33 | 88.89 |
| Crp3 | 91.67 | 97.22 |  | 44.74 | 58.33 | 88.89 |
| Crp4 | 44.74 | 47.37 | 50.00 |  | 47.37 | 39.47 |
| Crp5 | 66.67 | 69.44 | 69.44 | 55.26 |  | 55.56 |
| Crp6 | 94.44 | 88.89 | 88.89 | 44.74 | 69.44 |  |

The data on the upper right represent the sequence identity, and the data on the bottom left indicate the presence of sequence similarity.

**Additional file 2: Table s2.** Quantitative results of the amount of deformylation of Crps.

| Crps | Proportion (%) | | | |
| --- | --- | --- | --- | --- |
|  | Before defomylation | After deformyltion | | |
|  | Formyl-Crps | Formyl-Crps | **Crps** | By-product |
| Crp1 | 100 | 10 | **83** | 2 |
| Crp2 | 100 | 11 | **85** | 2 |
| Crp3 | 100 | 11 | **84** | 2 |
| Crp4 | 100 | 10 | **80** | 5 |
| Crp6 | 100 | 8 | **73** | 16 |

The results of Crps treated with 0 M (control) and 0.6 M HCl are shown in the table, and the results were confirmed by RP-HPLC. The proportion of each product was confirmed using the peak area of RP-HPLC.


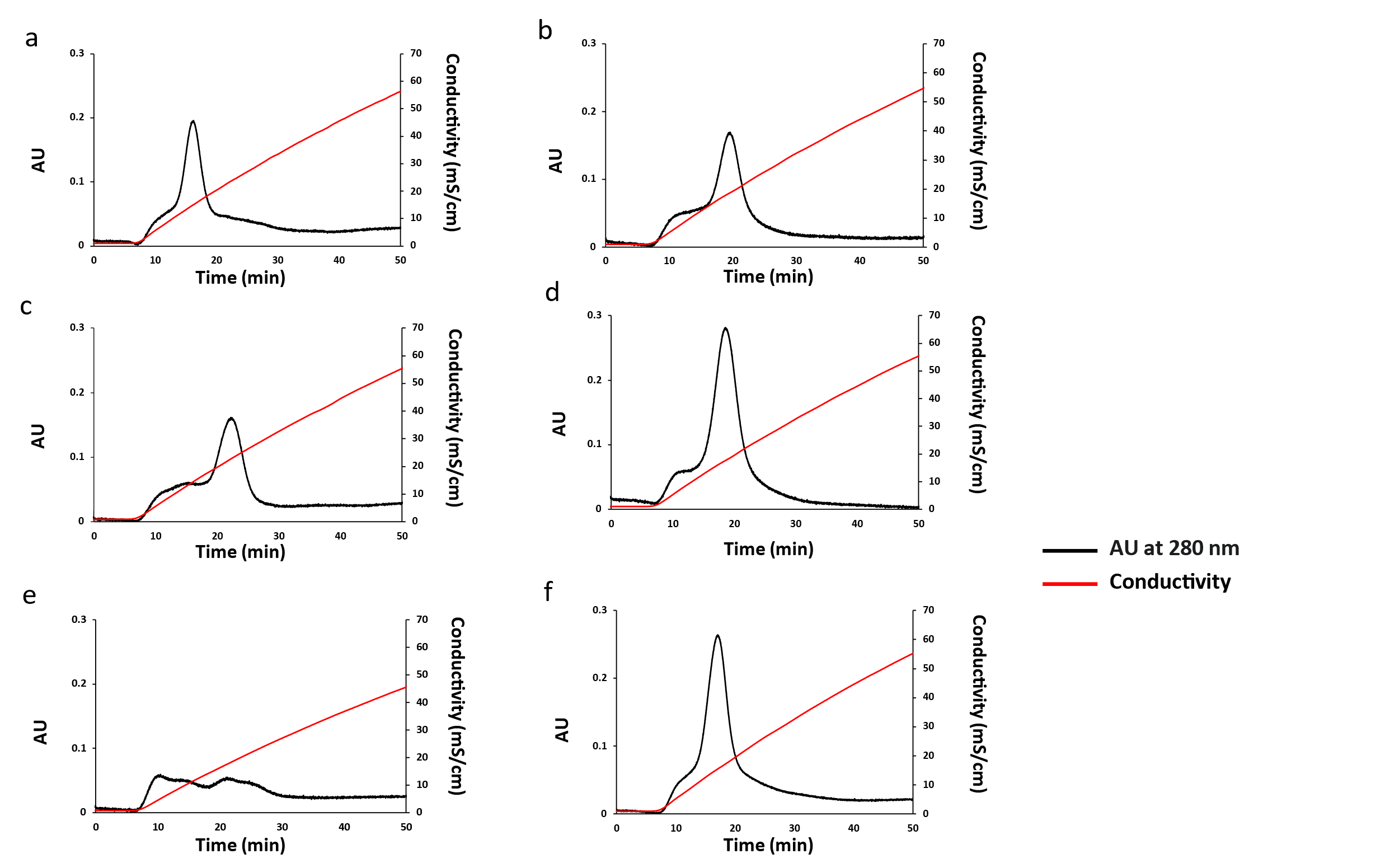


**Additional file 3: Fig. s1.** Cation exchange chromatography (CIEX) results for Crps. (a) Crp1; (b) Crp2; (c) Crp3; (d) Crp4; (e) Crp5; and (f) Crp6.


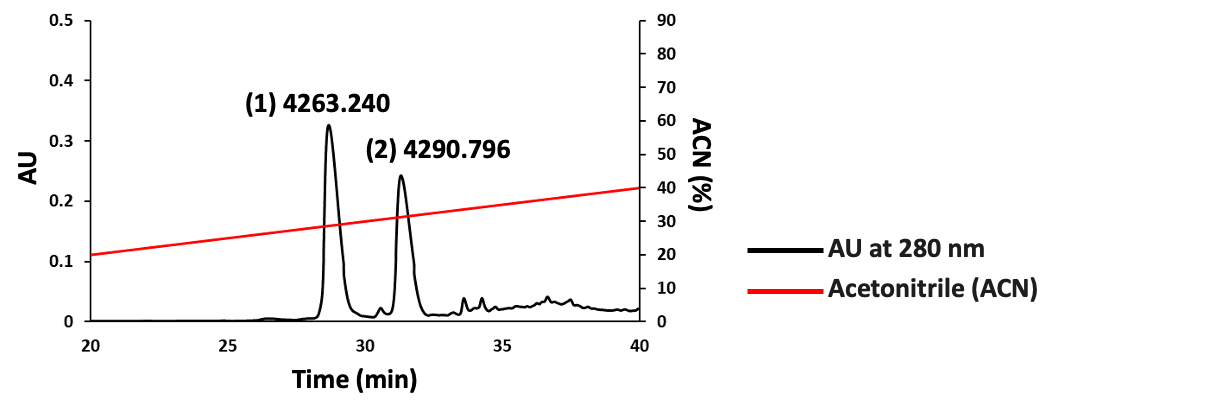


**Additional file 4: Fig. s2.** RP-HPLC results after purification of Crp6 using the BL21 strain. The two observable peaks are: (1) Crp6; and (2) formyl Crp6. The result for Crp6 by using the Origami™ B strain (Fig. 3e). The proportion of mature Crp6 was higher. Peptides produced in 1 L of medium were loaded.


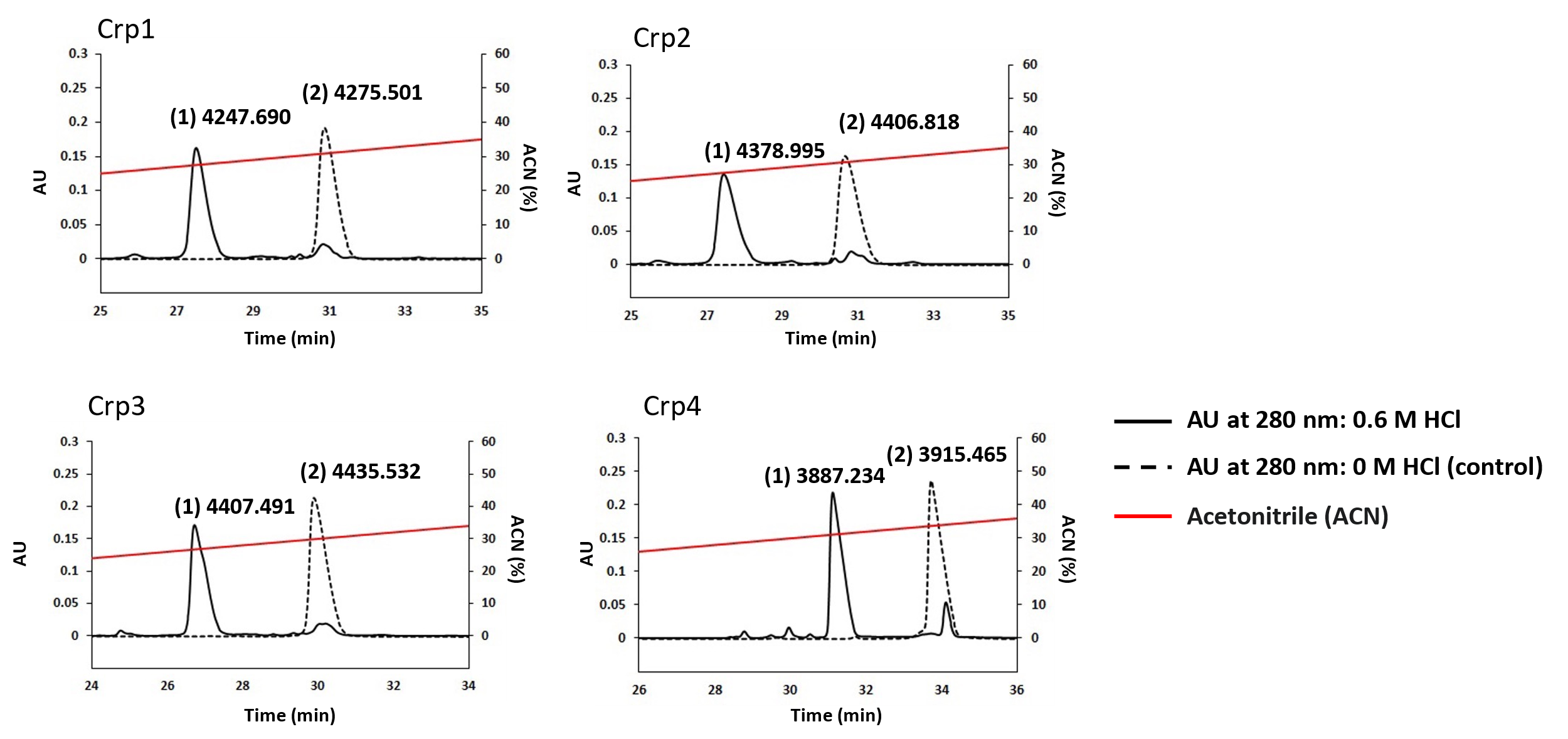


**Additional file 5: Fig. s3.** RP-HPLC results for Crp deformylation. The figure shows the results for Crps treated with 0 M(control) and 0.6 M HCl. The two observable peaks are: (1) Crps after deformylation; and (2) undeformylated Crps. The molecular weight of the mature Crps was determined using MALDI-TOF mass spectrometry. Approximately 100 μg of Crps were loaded.
